# Loss of AMPK activation promotes the invasion and metastasis of pancreatic cancer through an HSF1‐dependent pathway

**DOI:** 10.1002/1878-0261.12116

**Published:** 2017-08-29

**Authors:** Ke Chen, Weikun Qian, Jie Li, Zhengdong Jiang, Liang Cheng, Bin Yan, Junyu Cao, Liankang Sun, Cancan Zhou, Meng Lei, Wanxing Duan, Jiguang Ma, Qingyong Ma, Zhenhua Ma

**Affiliations:** ^1^ Department of Hepatobiliary Surgery First Affiliated Hospital Xi'an Jiaotong University China; ^2^ Department of Anesthesiology First Affiliated Hospital Xi'an Jiaotong University China

**Keywords:** AMP‐activated protein kinase, heat shock factor 1, invasion and metastasis, pancreatic cancer

## Abstract

Pancreatic ductal adenocarcinoma (PDAC) is a lethal malignancy with a mortality rate that closely parallels its incidence rate, and a better understanding of the molecular and cellular mechanisms associated with the invasion and distant metastasis is required. Heat shock factor 1 (HSF1) is a very highly conserved factor in eukaryotes that regulates the protective heat shock response. Here, we show that HSF1 is abnormally activated in pancreatic cancer. The knockdown of HSF1 impaired the invasion and migration and epithelial–mesenchymal transition (EMT) of pancreatic cancer cells *in vitro*; however, the upregulation of HSF1 showed the opposite effects. *In vivo*, the pharmacological inhibition of HSF1 significantly reduced the tumor burden, decreased the incidence of invasion, and prolonged the overall survival of transgenic mice harboring the spontaneous pancreatic cancer. We suggest that the loss of AMP‐activated protein kinase (AMPK) activation mediates the abnormal activation of HSF1 based on the findings that phospho‐HSF1 (p‐HSF1) was highly expressed in human PDAC tissues with a low expression of p‐AMPK and that in those tissues with a high p‐AMPK expression, the level of p‐HSF1 was decreased. The *in vivo* and *in vitro* activation of AMPK impaired the activity of HSF1, and HSF1 mediated the effects of the AMPK knockdown‐induced pancreatic cancer invasion and migration. Our study revealed a novel mechanism by which the loss of AMPK activation amplifies the activity of HSF1 to promote the invasion and metastasis of pancreatic cancer.

**Abbreviations**
ADM: acinar‐to‐ductal metaplasia
AMPK: AMP‐activated protein kinase
COX‐2: cyclooxygenase‐2
CSC: cancer stem cell
DMEM: Dulbecco's modified Eagle's medium
ECM: extracellular matrix
EMT: epithelial–mesenchymal transition
HGF: hepatocyte growth factor
HSF1: heat shock factor 1
MAPK: mitogen‐activated protein kinase
MDSC: myeloid‐derived suppressor cell
mTOR: mammalian target of rapamycin
NF‐κB: nuclear factor kappa B
PanIN: pancreatic intraepithelial neoplasia
PDAC: pancreatic ductal adenocarcinoma
PNI: perineural invasion
PSC: pancreatic stellate cell
SDF1: stroma‐derived factors 1
α‐SMA: α‐smooth muscle actin

## 1 Introduction

Pancreatic ductal adenocarcinoma (PDAC) is a lethal malignancy with a dismal prognosis (Siegel *et al*., 2017). Surgical resection is considered the most effective treatment for patients with PDAC. However, at the time of diagnosis, most patients have already developed to advanced stages and are not eligible for surgery (Conroy *et al*., 2011). Even following surgical resection, most patients die within two years of surgery because of recurrence and metastasis (Kamisawa *et al*., 2016). Gemcitabine has been considered the mainstay of chemotherapy for PDAC treatment, and recent studies have also revealed the therapeutic efficacy of gemcitabine combined with erlotinib (Moore *et al*., 2007), FOLFIRINOX (fluorouracil, folinic acid [leucovorin], irinotecan, and oxaliplatin) (Conroy *et al*., 2011), or nanoparticle albumin‐bound paclitaxel (nab‐paclitaxel) (Von Hoff *et al*., 2013). However, the overall 5‐year survival rate of patients with PDAC remains less than 8% (Siegel *et al*., 2017). A better understanding of the molecular and cellular mechanisms underlying the invasion and distant metastasis is required.

The progression of PDAC is associated with genetic and epigenetic alterations. Kras is one of the most commonly mutated genes in PDAC and plays a crucial role in the initiation and progression of PDAC; Kras mutations occur in more than 90% of patients with PDAC (Mann *et al*., 2016). Somatic mutations in the p53 tumor suppressor gene are also frequent genetic events that drive PDAC progression (Weissmueller *et al*., 2014). In addition, during the progression of PDAC, cancer cells establish a fertile microenvironment through complex interactions with stromal cells. Pancreatic stellate cells (PSCs) are resident cells that are frequently observed in PDAC tissues. Once activated, PSCs express high levels of α‐smooth muscle actin (α‐SMA), which is one of the markers of PSC activation, and then synthesize and secrete a large amount of extracellular matrix (ECM), thereby eventually contributing to the desmoplastic reaction in PDAC (Apte *et al*., 2013). The stroma of PDAC is also characterized by an infiltration of immune cells, including neutrophils, tumor‐associated macrophages, myeloid‐derived suppressor cells (MDSCs), and a small number of cytotoxic T cells (Sideras *et al*., 2014). Substantial evidence has revealed the role of the infiltrated neutrophils, macrophages, and MDSCs in invasion, metastasis, and the induction of an immunosuppressive microenvironment in PDAC (Felix and Gaida, 2016; Greten, 2014; Nielsen *et al*., 2016).

AMP‐activated protein kinase (AMPK) is considered a cellular metabolic stress sensor that plays a vital role in maintaining cellular energy homeostasis. By sensing the decreased adenosine monophosphate (AMP)/ATP level, AMPK was activated through Thr172 phosphorylation of the α‐subunit under metabolic stress conditions, such as starvation (Hardie, 2011; Stapleton *et al*., 1996). It has been suggested that AMPK acts as a tumor suppressor and impacts the tumor cell proliferation and cell cycle. Once activated, AMPK can influence several effectors, such as mammalian target of rapamycin, cyclooxygenase‐2 (COX‐2), and Akt, to exert its antitumor effect (Li *et al*., 2015). The epithelial‐to‐mesenchymal transition (EMT) is a complicated program that plays an important role in the invasion and metastasis of cancer (Carstens *et al*., 2014; Guo *et al*., 2016), and the EMT is characterized by the decreased expression of E‐cadherin, which is an epithelial marker, and the upregulation of mesenchymal markers, including N‐cadherin and vimentin (Du and Shim, 2016). Chou *et al*. (2014) revealed that the activation of AMPK is sufficient to reverse the mesenchymal phenotype of cancer cells partially through impairing the Akt‐MDM2‐Foxo3 signaling axis. The activation of AMPK was also shown to inhibit prostate cancer cell growth via the suppression of lipogenesis (Zadra *et al*., 2014). In pancreatic cancer, the role of AMPK remains to be fully elucidated.

Heat shock factor 1 (HSF1), which is highly conserved in eukaryotic species, is a transcriptional factor mediating the heat shock response. Under physiological conditions, HSF1 protects organisms and cells from various types of stress, such as heat, ischemia, oxidative stress, and other noxious conditions (Vihervaara and Sistonen, 2014). Several studies have demonstrated that HSF1 is activated and predicts a poor prognosis in various types of cancer, including breast cancer (Santagata *et al*., 2011), colon cancer (Jacobs and Marnett, 2009), and liver cancer (Li *et al*., 2014b). In cancer cells, HSF1 could drive a transcriptional program that is vital for supporting the malignant state of cancer, and the transcriptional program was highly enriched in protein translation, RNA binding, metabolism, and cell adhesion (Mendillo *et al*., 2012). However, little is known about the precise regulation of HSF1 in pancreatic cancer and its role in pancreatic progression.

Based on our previous finding that the loss of AMPK activation was a frequent event in pancreatic cancer (Duan *et al*., 2017), in the present study, we hypothesized that AMPK inactivation promotes the amplification and abnormal activation of the tumor‐promoting HSF1, thus promoting pancreatic cancer progression. We revealed a novel mechanism which might foster new therapeutic strategies for pancreatic cancer.

## 2 Materials and methods

### 2.1 KPC transgenic mice

Pdx1‐Cre mice, LSL‐Kras^G12D^ mice, and p53 ^fl/fl^ mice were purchased from the Nanjing Biomedical Research Institute of Nanjing University, Nanjing, China. The breeding of the LSL‐Kras^G12D/+^; p53 ^fl/+^; Pdx1‐Cre (KPC) mice was achieved by first crossing the p53 ^fl/fl^ mice with the Pdx1‐Cre mice to generate p53 ^fl/fl^; Pdx1‐Cre offspring. The p53 ^fl/fl^; Pdx1‐Cre mice were then crossed with the LSL‐Kras^G12D^ mice to generate the KPC animals. All mice were housed under pathogen‐free conditions and had free access to water and food. All experimental protocols were approved by the Ethical Committee of the First Affiliated Hospital of Medical College, Xi'an Jiaotong University, Xi'an, China.

### 2.2 Tissue preparation and histological analysis

We collected pancreatic cancer samples and normal pancreatic tissues from the Department of Hepatobiliary Surgery, the First Affiliated Hospital of Xi'an Jiaotong University after receiving approval from the Ethical Committee of Xi'an Jiaotong University. The pathologies of the pancreatic tissues were examined by two pathologists. The KPC mice were sacrificed, and the pancreas and other organs, such as the liver and lungs, were gently removed. The pancreatic tissues were weighed, and the tumor volumes were measured; then, the tissues were immediately fixed in 10% buffered formalin and embedded in paraffin. For the histopathological analysis, the tissues were sliced (5 μm), and hematoxylin and eosin (H&E) staining was performed according to the manufacturer's instructions. The liver and lungs were serially sectioned, and every fifth section was stained with H&E to detect distant metastasis. The identification of acinar‐to‐ductal metaplasia (ADM) and the grading of mPanIN (graded as mPanIN1A, mPanIN1AB, mPanIN2, and mPanIN3) and PDAC were based on previously described criteria (Hruban *et al*., 2006). The immunohistochemical staining was performed using a SABC Kit (Maxim, Fuzhou, China) according to the manufacturer's instructions. Briefly, the tissue sections were incubated with primary antibodies overnight at 4 °C and then incubated with the appropriate biotinylated secondary antibodies for 30 min at room temperature, followed by 30 min of incubation with streptavidin peroxidase (Dako LSAB+HRP Kit). After rinsing, the results were visualized using DAB, and the slides were counterstained with hematoxylin.

### 2.3 Cell culture and regents

Human Panc‐1, BxPC‐3, and MiaPaCa‐2 tumor cells were purchased from the Chinese Academy of Sciences Cell Bank of Type Culture Collection (CBTCCCAS, Shanghai, China). The Panc‐1 and MiaPaCa‐2 cell lines were cultured in Dulbecco's modified Eagle's medium (DMEM), and BxPC‐3 was cultured in RPMI‐1640, with 10% fetal bovine serum (HyClone, Logan, UT, USA), supplemented with 1% penicillin/streptomycin. The cells were cultured under standard conditions with a 5% CO_2_ atmosphere at 37 °C. Antibodies against various proteins were obtained from the following sources: HSF1, p‐HSF1, and α‐SMA were obtained from Abcam (Cambridge, MA, USA); HSP70, p‐AMPK, N‐cadherin, E‐cadherin, and vimentin were obtained from Cell Signaling Technology (Danvers, MA, USA); β‐actin was obtained from Sigma (St. Louis, MO, USA); and Ki67, CD68, myeloperoxidase (MPO), and CD3 were obtained from Servicebio (Wuhan, Hubei, China). Metformin and KRIBB11 were purchased from MCE (Greenville, SC, USA).

### 2.4 Quantitative real‐time PCR

The extraction of the total ribonucleic acid (RNA) was achieved using the Fastgen1000 RNA isolation system (Fastgen, Shanghai, China) according to the manufacturer's protocol. A Prime Script RT reagent kit (TaKaRa, Dalian, China) was used to reverse‐transcribe the total RNA into cDNA. Quantitative real‐time PCR was performed as previously described (Jiang *et al*., 2016). The PCR primer sequences used were as follows: HSF1 forward ACCCATGCTTCCTGCGTGGC, reverse TGCTTCTGCCGAAGGCTGGC; HSPH1, forward CACCAGAAAACCCAGACACT, reverse GGGAGACTGTGAGGTTTGTT; HSPA6, forward AGCAGTTGTGGCACTCAAG, reverse TCACAGCTGACTTATCACGAAG; HSPE1, forward ACACTAGAGCAGAGTACGAGTC, reverse CAGCACTCCTTTCAACCAATAC; and β‐actin, forward AGCGAGTATCCCCCAAAGTT, reverse GGGCACGAAGGCTCATCATT. The expression level of each target gene was determined using β‐actin as the normalization control. The relative gene expression was calculated using the 2^−ΔΔCt^ method.

### 2.5 Small interference RNA transfections

siRNA against HSF1 (HSF1‐Homo‐380: sense GCGGCAGCUCAACAUGUAUTT, antisense AUACAUGUUGAGCUGCCGCTT; HSF1‐Homo‐955: sense GACCCAUCAUCUCCGACAUTT, antisense AUGUCGGAGAUGAUGGGUCTT), AMPK (sense UUCUCCGAACGUGUCACGUTT, antisense ACGUGACACGUUCGGAGAATT), and a negative control siRNA (sense UUCUCCGAACGUGUCACGUTT, antisense ACGUGACACGUUCGGAGAATT) were purchased from GenePharm (Shanghai, China). Cells seeded in six‐well plates were transfected with 100 nm siRNA using the Lipofectamine RNAi MAX Reagent from Invitrogen (Carlsbad, CA, USA) according to the manufacturer's instructions. The cells were used in subsequent experiments 24 h after the transfection.

### 2.6 Western blot assays

Whole‐cell lysates of the Panc‐1 and BxPC‐3 cells were prepared using the RIPA buffer (Beyotime, Guangzhou, China) according to the manufacturer's instructions. The protein concentration was determined using a BCA protein assay kit (Pierce, Rockford, IL, USA). The protein lysates were resolved on a 10% polyacrylamide gel with a 5% stacking gel. The proteins were subsequently blotted on polyvinylidene difluoride membranes. The membranes were blocked for 2 h in TBS containing 0.1% (vol/vol) Tween‐20 and 10% (wt/vol) nonfat dry milk powder and then incubated with the primary antibodies overnight at 4 °C. Following the incubation with the secondary HRP‐coupled antibodies for 2 h at room temperature, the membranes were washed with 0.1% TBS/Tween‐20, and the immunocomplexes were detected using the enhanced chemiluminescence kit and a Molecular Imager ChemiDoc XRS System (Bio‐Rad Laboratories, Hercules, CA, USA). β‐Actin was used as the internal loading control.

### 2.7 Immunofluorescence analysis

The cells were fixed in 4% formaldehyde diluted in phosphate‐buffered saline (PBS) for 20 min. Following permeabilization with 0.3% Triton X‐100, the cells were treated with the blocking buffer (5% BSA in PBS) for 1 h and then incubated with the primary antibody at 4 °C overnight. Green‐conjugated secondary antibodies from Jackson ImmunoResearch Laboratories (West Grove, PA, USA) were used at room temperature, and the nuclei were stained with 4′‐6‐diamidino‐2‐phenylindole. Images were pseudocolored using a Zeiss Instruments confocal microscope.

### 2.8 Cell invasion and migration assays

A Matrigel invasion assay was performed as previously described to assess the invasive viability of pancreatic cancer cells (Zhang *et al*., 2016). In brief, the upper chambers of the wells were coated with Matrigel (BD Biosciences, Franklin Lakes, NJ, USA). The tumor cells (10^5^) were suspended in serum‐free DMEM and seeded into the upper chamber. The cells were allowed to invade toward the lower chamber, which contained DMEM with 10% fetal bovine serum (FBS), for 48 h and were then fixed with 4% formaldehyde, followed by the removal of the noninvasive cells in the upper chamber with a cotton swab. The invading cells were stained with 0.1% crystal violet. The invading cell numbers were quantified by counting the stained cells under a microscope. The migration ability of the cancer cells was assessed by wound‐healing assays. The cancer cells were pretreated with NC‐siRNA, HSF1‐siRNA, AMPK‐siRNA, heat stress (HS) (42 °C, 1 h), or HSF1‐siRNA, followed by HS or AMPK‐siRNA, followed by BRIBB11, and then, the cells were scratched using a 10‐μL pipette tip. Next, the cancer cells were washed three times with PBS and cultured in a serum‐starved medium. Images were obtained under a microscope from Nikon Instruments Inc. (Shanghai, China).

### 2.9 Statistical analysis

The data are presented as the mean ± SD. Comparisons between groups were analyzed by Student's *t*‐test using spss (version 15.0; SPSS, Chicago, IL, USA). Kaplan–Meier analysis was used for the survival analysis. *P*‐values < 0.05 were considered significant.

## 3 Results

### 3.1 Activation of HSF1 is an early event in pancreatic cancer and correlates with the inactivation of AMPK

**Figure 1:**
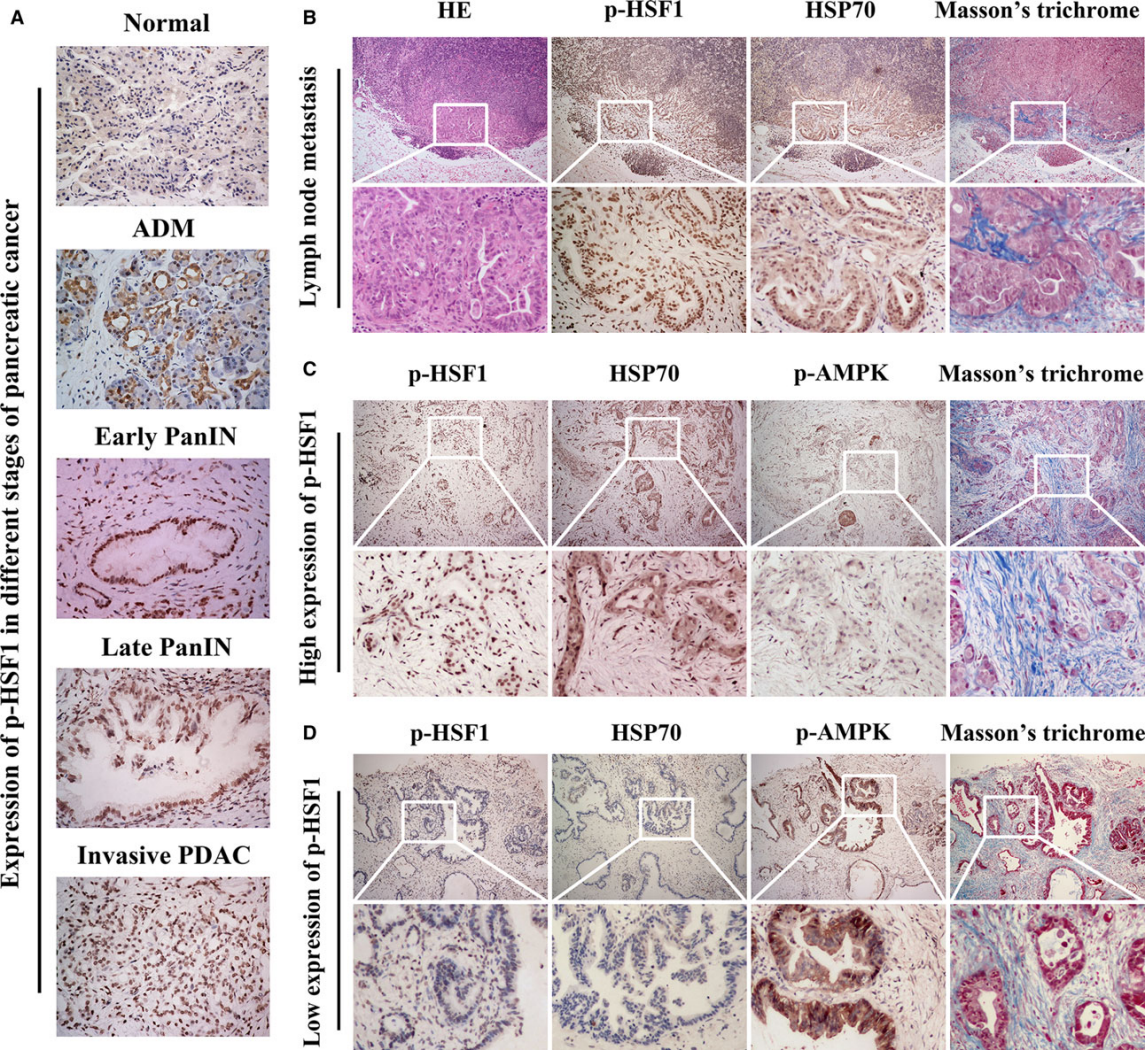
Pancreatic cancer showed an elevated expression of p‐HSF1 but a loss of AMPK activation. (A) Representative images showing the expression of p‐HSF1 in normal pancreatic tissue, ADM, early PanIN, late PanIN, and invasive PDAC. (B) Representative images showing H&E staining, IHC staining of p‐HSF1 and HSP70, and Masson's staining of collagen in tissues from lymph node metastasis. (C) Images showing the expression of p‐AMPK and Masson‐stained collagen in tissues with a high expression of p‐HSF1. (D) Images showing the expression of p‐AMPK and Masson‐stained collagen in tissues with a low expression of p‐HSF1. Scale bars = 100 μm.

To investigate the role of HSF1 and AMPK in PDAC, we firstly analyzed the expression of phospho‐HSF1 (p‐HSF1) and phospho‐AMPK (p‐AMPK) in human PDAC tissues. We found that p‐HSF1 was weakly detected in the normal pancreatic tissues. p‐HSF1 staining was observed in the pancreatic tissues undergoing ADM. In pancreatic intraepithelial neoplasia (PanIN), including early and late PanIN, we observed strong nuclear staining of p‐HSF1, indicating its activation, phosphorylation, and nuclear translocation in precursor lesions of PDAC (Fig. 1A). To investigate the role of HSF1 in pancreatic cancer invasion and metastasis, we detected the expression of p‐HSF1 in tissues of invasive PDAC and lymph node metastasis. A high expression of p‐HSF1 was detected in the invasive PDAC tissues (Fig. 1A). In the lymph node metastatic tissues, we observed strong IHC staining of p‐HSF1 and one of the downstream targets, HSP70 (Fig. 1B), which has been implicated in carcinogenesis and the progression of various types of cancers (Cho *et al*., 2012). In addition, we observed a deposition of Masson‐stained collagen I in the lymph node metastatic tissue (Fig. 1B). Interestingly, we found that p‐AMPK was weakly detected in the PDAC tissues with a high level of p‐HSF1 (Fig. 1C). In contrast, in tissues with a low level of p‐HSF1, p‐AMPK was strongly expressed (Fig. 1D). These results suggested that the activation of HSF1 is an early event in pancreatic cancer. The expression and activation of HSF1 is associated with the inactivation of AMPK.

### 3.2 Transgenic engineered mice recapitulated the expression of HSF1 and AMPK in human PDAC tissues

**Figure 2:**
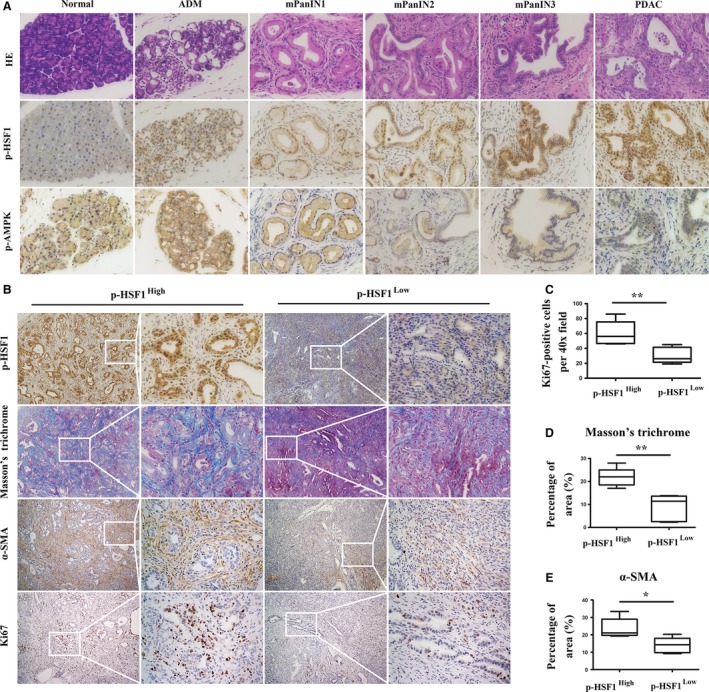
Genetically engineered mice recapitulated the expression of p‐HSF1 and p‐AMPK in human PDAC tissues. (A) Representative images showing the expression of p‐HSF1 and p‐AMPK in mouse normal pancreatic tissues, ADM, mPanIN1, mPanIN2, mPanIN3, and invasive PDAC. (B) Representative images showing IHC staining of HSF1, Masson‐stained collagen, and IHC staining of α‐SMA and Ki67 in tissues with different levels of p‐HSF1. (C) Quantification of Ki67 in pancreatic tissues between the low and high p‐HSF1 groups. (D, E) Quantification of Masson's trichrome and α‐SMA between the low and high p‐HSF1 groups. Scale bars = 100 μm. *P < 0.05, **P < 0.01.

To further investigate the role of AMPK and HSF1 in PDAC, we generated LSL‐Kras^G12D/+^; p53 ^fl/+^; Pdx1‐Cre (KPC) transgenic mice that recapitulate the spectrum of pancreatic cancer from pancreatic intraepithelial neoplasia (PanIN) to invasive PDAC (Lee *et al*., 2016). First, immunohistochemical staining was performed to detect the expression of p‐HSF1 and p‐AMPK in different stages of pancreatic cancer. We found that in the normal pancreas, p‐HSF1 was weakly detected in the cytoplasm of acinar cells. In ADM and mouse pancreatic intraepithelial neoplasia 1 (mPanIN1), the expression of p‐HSF1 was elevated (Fig. 2A). p‐HSF1 was significantly upregulated in late mPanIN (mPanIN2 and mPanIN3), in which the flat epithelium turned to papillary structures accompanied by nuclear abnormalities, including the loss of polarity and nuclear crowding, and invasive PDAC (Fig. 2A). p‐AMPK was detected in the normal pancreatic tissue, ADM and mPanIN1. Interestingly, we found that the expression of p‐AMPK was downregulated as the disease progressed from PanIN1 to PanIN3. In invasive PDAC, p‐AMPK showed a relatively low expression level (Fig. 2A).

We then performed Masson's trichrome staining to detect the deposition of collagen and fibrils in the pancreatic tissues (Fig. 2B). We found that high expression of p‐HSF1 was associated with increased deposition of collagen (Fig. 2D). IHC staining of α‐SMA showed that α‐SMA was uniformly expressed in pancreatic tissues with a high p‐HSF1 level. However, in pancreatic tissues with a low expression of p‐HSF1, α‐SMA was weakly detected (Fig. 2B,E). We also revealed a correlation between HSF1 and the proliferation index Ki67 (Fig. 2B,C). These data suggested that as pancreatic cancer progresses from ADM and low‐grade PanIN1 to high‐grade PanIN3 and then eventually to invasive PDAC, HSF1 was gradually activated; however, the expression of p‐AMPK showed the opposite pattern. The activation of HSF1 was associated with desmoplasia and cellular proliferation.

### 3.3 HSF1 promoted the invasion and migration of pancreatic cancer cells

**Figure 3:**
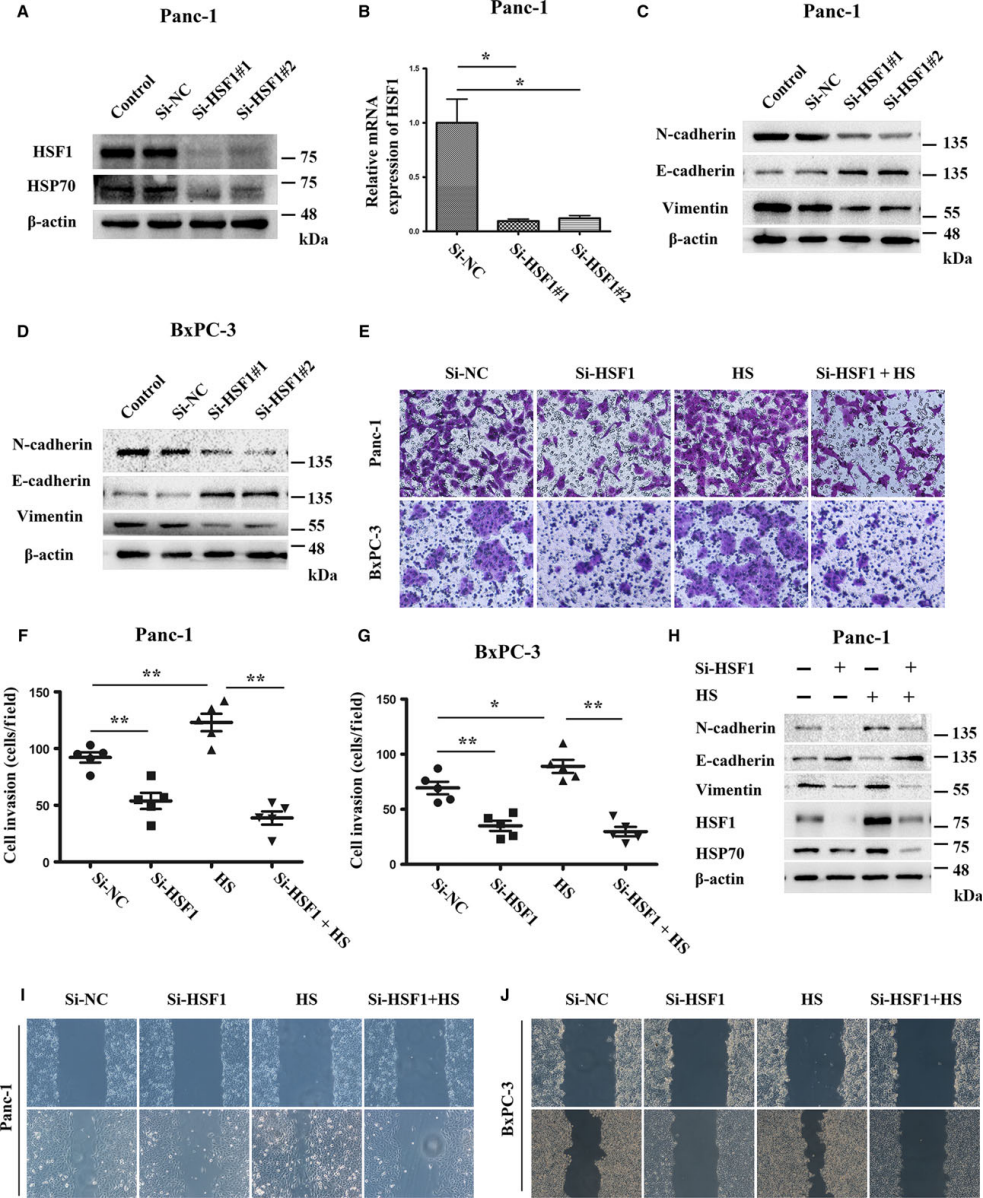
HSF1 promoted the invasion and migration of pancreatic cancer cell lines. (A, B) Western blotting and quantitative real‐time PCR showing the knockdown of HSF1 by HSF1‐siRNA in the Panc‐1 cells. (C, D) Western blotting assays were performed to evaluate the effect of HSF1‐siRNA on the protein expression of N‐cadherin, E‐cadherin, and vimentin in the Panc‐1 and BxPC‐3 cells. (E) Panc‐1 and BxPC‐3 cells were pretreated with HSF1‐siRNA, heat stress (HS) (42 °C, 1 h), or HSF1‐siRNA, followed by HS, and then, the cells were seeded into Matrigel‐coated invasion chambers for an additional 48 h to evaluate the invasive viability. (F, G) Quantification of the invasive cells in different groups treated as indicated. (H) Western blotting assays were performed to detect the expression of p‐HSF1, HSP70, N‐cadherin, E‐cadherin, and vimentin in Panc‐1 cells treated with HSF1‐siRNA, HS, or HSF1‐siRNA, followed by HS. (I, J) The migration capacity of the Panc‐1 and BxPC‐3 cells in response to HSF1‐siRNA, HS, or HSF1‐siRNA, followed by HS, was detected using wound‐scratch assays, and the images were visualized after 0 and 36 h at a magnification of 40 ×. *P < 0.05, **P < 0.01.

Next, we investigated the role of HSF1 in the invasion and migration of pancreatic cancer. The expression of HSF1 was downregulated in Panc‐1 cells by the HSF1‐specific small interference RNA (Fig. 3A,B). The Matrigel invasion assay results showed that the downregulation of HSF1 by the HSF1‐siRNA significantly inhibited the invasion ability of the pancreatic cancer cells (PCs), and we reconfirmed this result in another pancreatic cancer cell line, BxPC‐3 (Fig. 3E). Furthermore, the PC migration ability was also impaired after the HSF1 downregulation, as demonstrated by the wound‐scratch assays (Fig. 3I,J). In cancer cells, the EMT confers the ability to invade the basement membranes and spread to the vasculature or lymphatic duct to form distant metastasis. We then investigated whether HSF1 could affect the EMT process. Western blotting verified that in both the Panc‐1 and BxPC‐3 cell lines, the downregulation of HSF1 resulted in a marked decrease in the expression of N‐cadherin and vimentin and a significant increase in E‐cadherin (Fig. 3C,D).

To further elucidate the important role of HSF1 in the invasion and migration of PCs, PCs were heat‐stressed (42 °C, 1 h) to increase the expression of HSF1. After 24 h of recovery, the invasion and migration abilities of the PCs were re‐evaluated. We observed that the HS definitively induced the expression of HSF1 and its downstream target, HSP70. In addition, we found that the HS upregulated the expression of N‐cadherin and vimentin but downregulated the expression of E‐cadherin (Fig. 3H). Furthermore, the HS increased the invasion and migration of PCs (Fig. 3E,I,J). To confirm that the HS‐induced invasion and migration of PCs were dependent on the activation of HSF1, the PCs were pretreated with Si‐HSF1 and then treated with HS. We found that when pretreated with Si‐HSF1, HS failed to induce the invasion and migration of PCs and the EMT process (Fig. 3E,H–J). Altogether, these data suggested that HSF1 participates in the invasion and migration of pancreatic cancer and the EMT process.

### 3.4 HSF1 inhibition suppressed the progression of pancreatic cancer *in vivo*

**Figure 4:**
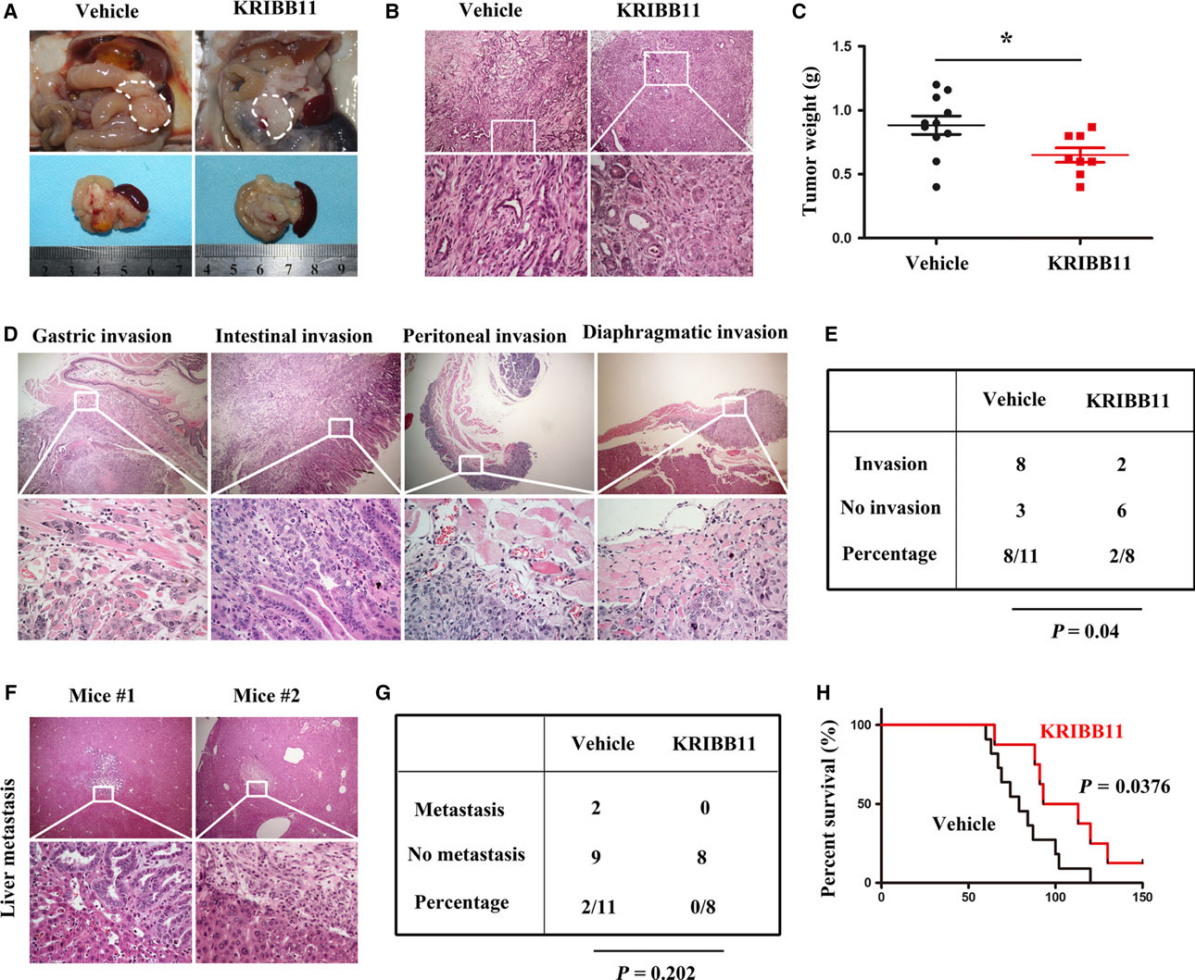
*In vivo* inhibition of HSF1 reduced the tumor burden and suppressed the invasion of pancreatic cancer. (A) Representative macroscopic images of PDAC in KPC mice treated with vehicle or KRIBB11. The white dotted line shows the pancreas. (B) Histology of tumors from KPC mice in groups as indicated. (C) Quantification of the tumor weight in mice treated with vehicle or KRIBB11. (D) Representative images showing gastric wall invasion, intestinal invasion, peritoneal invasion, and diaphragmatic invasion in KPC mice. (E) Table listing the incidence of invasion in the KPC mice that were treated with vehicle or KRIBB11. (F) HE staining showing that liver metastasis occurred in the mice treated with vehicle. (G) Table listing the incidence of distant metastasis in the KPC mice treated with vehicle or KRIBB11. (H) Kaplan–Meier survival analysis of KPC mice treated with vehicle or KRIBB11. Scale bars = 100 μm. *P < 0.05.

To further investigate whether HSF1 inhibition could inhibit the progression of pancreatic cancer, starting at 8 weeks of age, when KPC mice have developed widespread advanced pancreatic neoplasia, we treated the KPC mice daily with vehicle or KRIBB11 (N(2)‐(1H‐indazole‐5‐yl)‐N(6)‐methyl‐3‐nitropyridine‐2,6‐diamine) (50 mg·kg^−1^), which is a potent HSF1 inhibitor (Yoon *et al*., 2011). Compared to the mice that were treated with the vehicle, we found that the treatment with KRIBB11 achieved a longer overall survival (Fig. 4H). Most vehicle‐treated KPC mice developed widespread tumors involving the whole pancreas; however, in those treated with KRIBB11, the tumors were localized in the head or tail of the pancreas, and the remaining pancreatic tissues were normal (Fig. 4A). The histological analysis showed that the tumors in the mice treated with the vehicle presented dense fibrotic stroma interspersed with moderate to poor differentiated cancer cells and areas of necrosis. We observed that those treated with KRIBB11 showed well to moderately differentiated cancer cells; In addition, we found residual acinus and low‐grade duct‐like structures within the tumors (Fig. 4B). Accordingly, the mice treated with KRIBB11 had a decreased tumor weight (Fig. 4C).

Among those treated with the vehicle, we observed abdominal invasions, including gastric wall invasion, intestinal invasion, peritoneal invasion, and diaphragmatic invasion (Fig. 4D). We found that the KRIBB11 treatment dramatically decreased the incidence of abdominal invasions; only one of eight mice showed intestinal invasion, and one mouse showed peritoneal invasion (Fig. 4E). Hepatic metastasis was observed in two of the 11 vehicle‐treated mice, and we did not observe hepatic metastasis in the mice treated with KRIBB11 (Fig. 4F,G). Collectively, these data suggest that HSF1 inhibition decreased the tumor burden and abdominal invasion. Importantly, the inhibition of HSF1 prolonged the overall survival of the KPC mice.

### 3.5 AMPK activation suppressed the activity of HSF1

**Figure 5:**
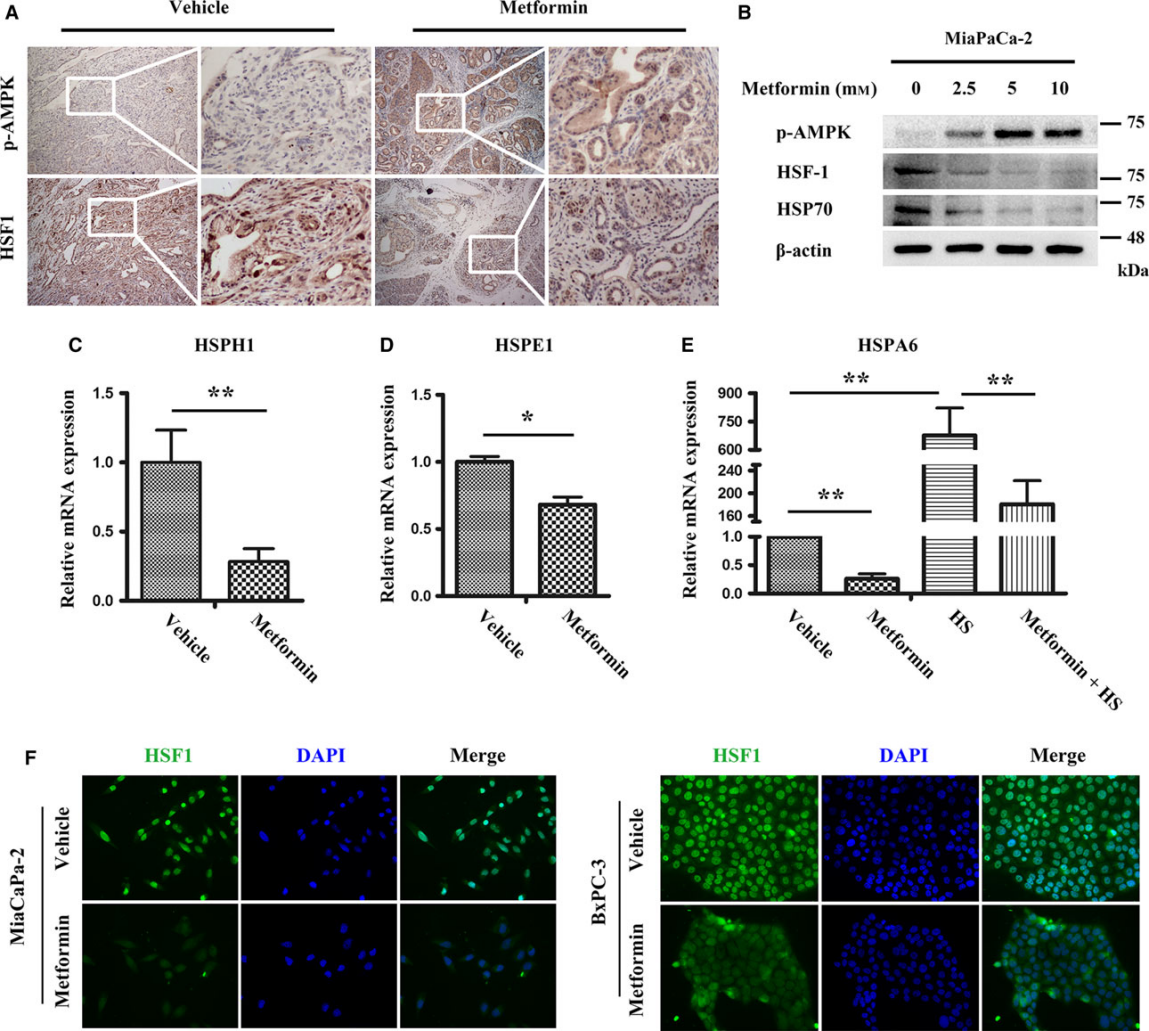
The activity of HSF1 was suppressed by the metformin‐mediated AMPK activation. (A) Representative IHC images showing the expression of p‐AMPK and HSF1 in KPC mice treated with vehicle or metformin. Scale bars = 100 μm. (B) MiaPaCa‐2 cells were treated with a gradient concentration of metformin for 24 h, and then, western blotting was performed to show the activation of p‐AMPK and the suppression of HSF1 and HSP70. (C, D) MiaPaCa‐2 cells were pretreated with vehicle or metformin (5 mm) for 24 h, and then, quantitative real‐time PCR was performed to detect the transcription of HSPH1 and HSPE1. (E) Cells were pretreated with metformin (5 mm) for 24 h, followed by HS (42 °C, 1 h), and then, the cells were recovered at 37 °C for 6 h; Quantitative real‐time PCR showing that metformin inhibited the HS‐induced HSFA6 transcription. (F) Immunofluorescence showing the expression of HSF1 in MiaPaCa‐2 and BxPC‐3 cells treated as indicated. Scale bars = 50 μm. *P < 0.05, **P < 0.01.

Because the high expression of HSF1 was associated with a low expression of p‐AMPK, we wondered whether interventions targeting AMPK could impact the activity of HSF1. To verify our hypothesis, IHC of p‐AMPK and HSF1 was performed. In pancreatic tissue from mice treated with metformin, the expression of p‐AMPK was upregulated, but the expression of HSF1 was decreased (Fig. 5A).

Next, an *in vitro* study was conducted to further explore the effect of AMPK activation on HSF1 suppression. We cultured three pancreatic cancer cell lines, MiaPaCa‐2, Panc‐1, and BxPC‐3. After starvation in FBS‐free DMEM for 8 h, the MiaPaCa‐2 and Panc‐1 cells were treated with gradient concentration of metformin (0, 2.5, 5, 10 mm) for 24 h; then, the cellular total protein was extracted. Western blotting was performed to detect the expression of HSF1 and its downstream target. As expected, the gradient concentration of metformin induced the activation of AMPK, which was determined by its increased phosphorylation. The expression of HSF1 and HSP70 was both downregulated upon the metformin treatment. In addition, we found that metformin at a concentration of 5 mm is sufficient to induce the activation of AMPK and the suppression of HSF1 (Figs 5B, S1A). To further investigate the effect of AMPK activation on HSF1, we treated the MiaPaCa‐2 cells with 5 mm metformin. Total mRNA was extracted 24 h after the treatment, and quantitative real‐time PCR was conducted to detect the transcription of HSPH1, HSPE1, and HSPA6, which are three characterized targets of HSF1. We revealed that the metformin treatment significantly downregulated the transcription of HSPH1, HSPE1, and HSPA6 (Fig. 5C–E). Because HSF1 is sensitive to HS, we aimed to investigate whether AMPK activation could impact the activity of HSF1 under heat shock stress. The MiaPaCa‐2 cells were pretreated with 5 mm metformin for 24 h and then heat‐shocked at 42 °C for 1 h. After six hours of recovery at 37 °C, we monitored the transcriptional level of HSFA6. The HS induced a tremendous upregulation of the transcription of HSFA6; however, the transcription of HSFA6 was significantly reduced following the metformin treatment (Fig. 5E).

The activation of HSF1 involved its phosphorylation, trimerization, and translocation from the cytoplasm to the nucleus. Thus, we investigated whether the metformin‐induced AMPK activation could influence the nuclear translocation of HSF1. The MiaPaCa‐2, Panc‐1, and BxPC‐3 cell lines were treated with metformin for 24 h, and then, immunofluorescence was performed to detect the expression of HSF1. We detected high levels of HSF1, particularly in the nucleus. Upon the metformin treatment, the fluorescence intensity in the nucleus was decreased, indicating an impairment in the nuclear translocation of HSF1 (Figs 5F, S1B). These data suggested that AMPK activation suppressed the activity of HSF1 by impairing its phosphorylation and nuclear translocation.

### 3.6 HSF1 was required for the AMPK inactivation‐mediated pancreatic cancer invasion and migration

**Figure 6:**
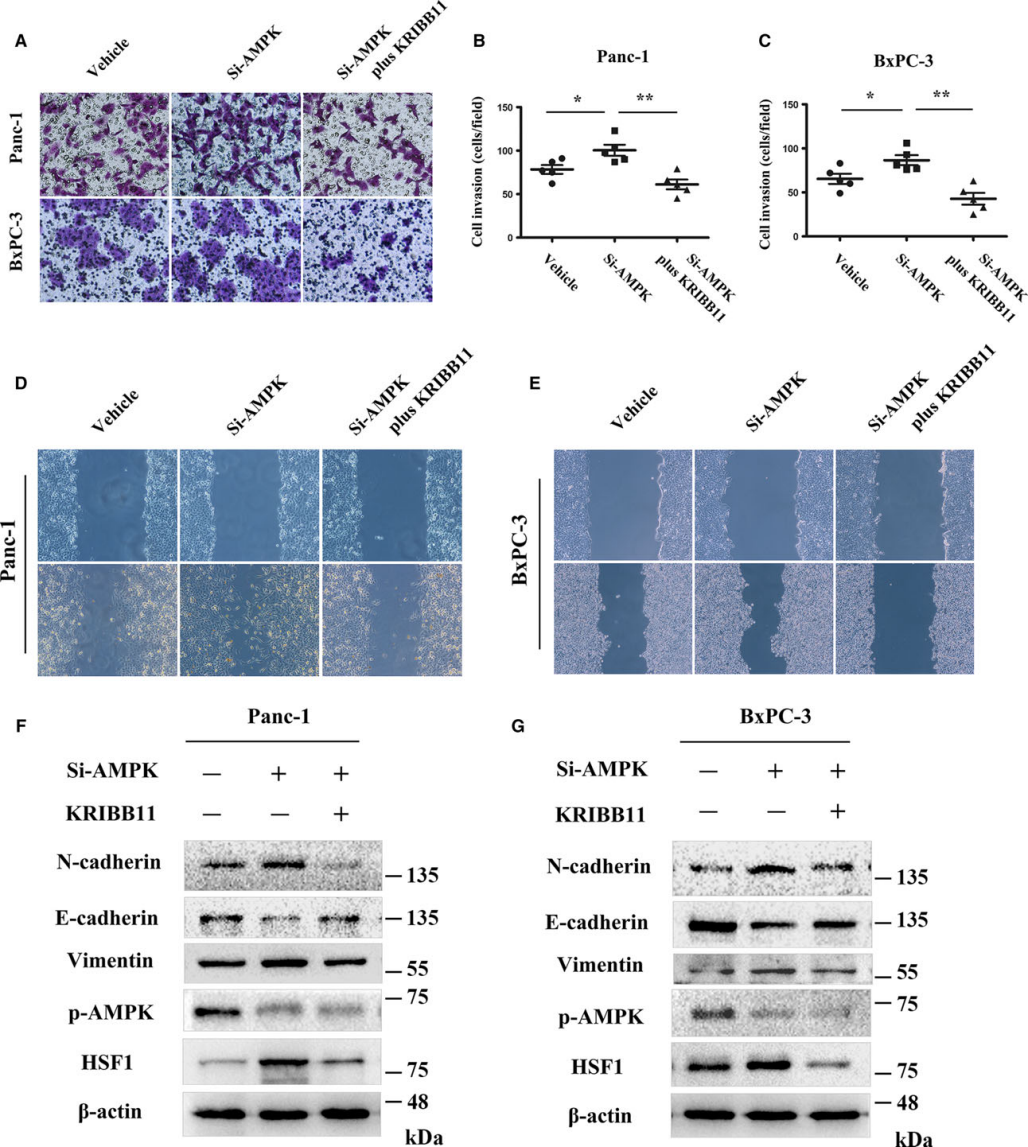
HSF1 is required for the AMPK inactivation‐induced invasion and migration of pancreatic cancer cells. (A) Matrigel invasion assays were performed to assess the invasive ability of Panc‐1 and BxPC‐3 cells treated with vehicle, AMPK‐siRNA, or AMPK‐siRNA, followed by KRIBB11. (B, C) Quantification of the invasive cells in different groups treated as indicated. (D, E) The migration capacity of Panc‐1 and BxPC‐3 cells in response to vehicle, AMPK‐siRNA, or AMPK‐siRNA, followed by KRIBB11 treatment, was detected using wound‐scratch assays, and the images were visualized after 0 and 36 h at a magnification of 40 ×. (F, G) Western blotting assays were performed to detect the expression of p‐AMPK, HSF1, N‐cadherin, E‐cadherin, and vimentin in Panc‐1 and BxPC‐3 cells treated with vehicle, AMPK‐siRNA, or AMPK‐siRNA, followed by KRIBB11. *P < 0.05, **P < 0.01.

Previous studies have suggested that AMPK plays a role in regulating the invasion and metastasis of cancer and the EMT process (Chou *et al*., 2014; Duan *et al*., 2017). We aimed to further investigate the role of AMPK in pancreatic cancer invasion and migration. As expected, the siRNA‐mediated knockdown of AMPK enhanced the invasion of the Panc‐1 and BxPC‐3 cell lines (Fig. 6A–C). The wound‐scratch assays also showed an increased migration of cancer cells (Fig. 6D,E). The western blotting showed that the AMPK knockdown upregulated the expression of N‐cadherin and vimentin; however, the expression of E‐cadherin was downregulated, indicating that AMPK inactivation plays an important role in the EMT process (Fig. 6F,G).

The western blotting showed that the knockdown of AMPK upregulated the expression of HSF1 (Fig. 6F,G). Given the previous result that metformin‐mediated AMPK activation suppressed the activity of HSF1, we wondered whether HSF1 is required for the AMPK inactivation‐mediated pancreatic cancer invasion and metastasis. To verify this hypothesis, Panc‐1 and BxPC‐3 cells were treated with KRIBB11 to inhibit the activity of HSF1 after the knockdown of AMPK by Si‐AMPK. Interestingly, we found that the inhibition of HSF1 significantly inhibited the AMPK inactivation‐mediated invasion and migration of the pancreatic cells. In addition, the HSF1 inhibition reversed the effect of the AMPK knockdown in the EMT process (Fig. 6A,D–E). These results indicated that HSF1 is required for the AMPK inactivation‐mediated promotion of pancreatic cancer invasion and migration.

### 3.7 HSF1 inhibition recapitulated the effects of the AMPK activation *in vivo*

**Figure 7:**
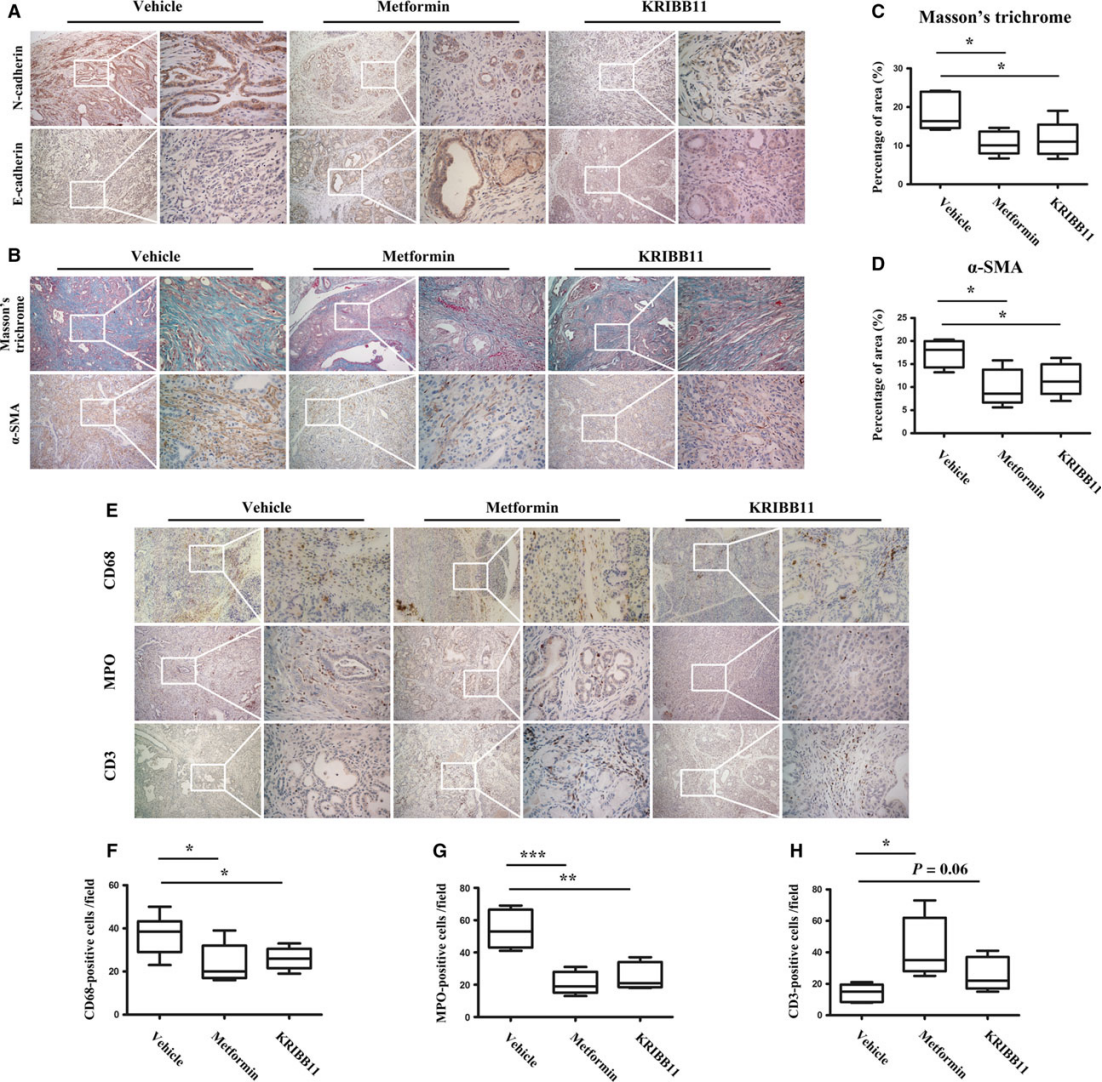
HSF1 inhibition recapitulated the effect of the metformin‐mediated AMPK activation. (A) IHC staining of N‐cadherin and E‐cadherin in the pancreas from KPC mice treated with vehicle, metformin, or KRIBB11. (B) Masson's trichrome staining and IHC staining of α‐SMA in pancreatic tissues from mice treated with vehicle, metformin, or KRIBB11. (C) Quantification of Masson's trichrome in mice as indicated. (D) Quantification of α‐SMA in mice as indicated. (E) IHC staining of CD68, MPO, and CD3 in KPC mice treated with vehicle, metformin, or KRIBB11. (F–H) Quantification of CD68‐positive cells (F), MPO‐positive cells (G), and CD3‐positive cells (H) in tumors from KPC mice as indicated. Scale bars = 100 μm. *P < 0.05, **P < 0.01, ***P < 0.001.

Because the AMPK activation suppressed the activity of HSF1, we aimed to investigate whether the HSF1 inhibition could recapitulate the effects of AMPK activation *in vivo*. IHC to detect the EMT‐associated markers was performed, and we found that in pancreatic tissues from KPC mice treated with metformin or KRIBB11, the expression of N‐cadherin was downregulated; however, the expression of the epithelial marker E‐cadherin was elevated (Fig. 7A). This result indicated that the HSF1 inhibition had a similar effect on the EMT *in vivo*.

We previously reported that in an orthotopic model of pancreatic cancer, AMPK activation leads to a reduced fibrogenic cytokine production and resulted in the inhibition of PSC activation (Duan *et al*., 2017). Thus, we aimed to investigate whether AMPK activation could impact the microenvironment of pancreatic cancer in KPC transgenic mice. Unsurprisingly, the treatment with metformin reduced the staining of Masson's trichrome and the expression of α‐SMA (Fig. 7B). We found that the KRIBB11 treatment also decreased the staining of Masson's trichrome and the expression of α‐SMA (Fig. 7B–D), suggesting that the AMPK‐mediated HSF1 inhibition played a role in suppressing the desmoplastic reaction of pancreatic cancer.

Substantial evidence has supported the notorious role of the inflammatory microenvironment in pancreatic cancer (Felix and Gaida, 2016; Nielsen *et al*., 2016; Wang *et al*., 2017). We then investigated the role of AMPK and HSF1 in the modulation of the inflammatory microenvironment of pancreatic cancer. Unsurprisingly, we found high numbers of MPO‐positive cells in the stroma of pancreatic cancer, which are indicative of the infiltration of neutrophils or their precursors. In addition, we also found high numbers of CD68‐positive macrophages. However, few CD3‐positive T cells were observed, which is indicative of the immunosuppressive characteristic of the microenvironment (Fig. 7E). We found that in the KPC mice that were treated with metformin or KRIBB11, there was a significant reduction in MPO‐positive neutrophils and CD68‐positive macrophages (Fig. 7F,G). More importantly, the treatment with metformin or KRIBB11 induced the recruitment of CD3‐positive T cells into the stroma (Fig. 7H). These findings highlight the important role played by the AMPK‐mediated HSF1 suppression in modulating the pancreatic desmoplastic reaction and inflammatory microenvironment.

## 4 Discussion

PDAC is a notoriously aggressive malignancy with a dismal prognosis, and a full understanding of its underlying mechanisms is required (Siegel *et al*., 2017). Here, we showed that HSF1 was abnormally activated in pancreatic cancer; however, AMPK was uniformly inactivated. The reproducibility of the tumor onset and progression in the genetically engineered KPC mouse model has allowed us to explore how AMPK and HSF1 affect the progression of PDAC. We revealed that the KPC mice recapitulated the expression of HSF1 and AMPK observed in human PDAC tissues. HSF1 was abnormally upregulated as pancreatic cancer progressed from low‐grade PanIN1 to invasive PDAC; however, the expression of AMPK showed the opposite pattern. We found that HSF1 promoted the pancreatic cancer invasion and metastasis; the *in vivo* study showed that the inhibition of HSF1 significantly reduced the tumor burden, suppressed the invasion, and prolonged the overall survival. The AMPK inactivation promoted the invasion and migration of pancreatic cancer cells in an HSF1‐dependent manner; the metformin‐mediated AMPK activation suppressed the activity of HSF1 and, thus, inhibited the EMT process, suppressed the desmoplastic reaction, and alleviated the inflammatory microenvironment.

HSF1 has been implicated in tumorigenesis and the progression of several cancers (Mendillo *et al*., 2012). Mendillo *et al*. (2012) revealed that in addition to its indirect action of inducing the expression of heat shock proteins, such as HSP90 and HSP70, HSF1 could drive a transcriptional program that is different from heat shock to facilitate the progression of malignant cancers. Previous studies have reported that the activation of HSF1 promoted the growth of hepatocellular carcinoma by stimulating lipid biosynthesis and activating the nuclear factor kappa B (NF‐κB) signaling pathway and mitogen‐activated protein kinase (MAPK) signaling pathway (Chuma *et al*., 2014; Jin *et al*., 2011). In breast cancer, nearly 80% of *in situ* and invasive breast carcinomas expressed high levels of HSF1, which was associated with a high histological grade, a larger tumor size, and nodal involvement; those with HSF1‐positive tumors had significantly reduced survival rates compared with women with HSF1‐negative tumors (Santagata *et al*., 2011). A subsequent study revealed that in breast cancer cell lines, the overexpression of HSF1 induced an augmentation of the cancer stem cell markers and conferred resistance to chemotherapeutic drugs (Wang *et al*., 2015). Here, we showed that pancreatic cancer expressed a high level of HSF1, which is similar to other cancers. In addition, the high expression of HSF1 in PDAC was associated with a dense desmoplastic reaction and increased cellular proliferation. The *in vitro* and *in vivo* studies also highlighted the promising therapeutic effect through the inhibition of HSF1.

The mechanism by which HSF1 is precisely regulated in cancer remains to be fully elucidated. A previous study has reported that the loss of the tumor suppressor gene neurofibromatosis type 1 (Nf1) triggered the activation of HSF1 via the MAPK/ERK signaling pathway. The authors found that in cell lines from human malignant peripheral nerve sheath tumors driven by Nf1 loss, HSF1 was overexpressed, phosphorylated, and translocated to the nucleus (Dai *et al*., 2012). Li *et al*. (2014a) reported that a mutation in p53 could act as a promoter of HSF1. Mutant p53 (mutp53) proteins stimulated the EGFR/ErbB2/MAPK/PI3K signaling cascades and, thus, promoted the phosphorylation and stabilization of HSF1; in turn, the activation of HSF1 stabilized the expression of mutp53 by the transcription of HSP90, hence forming a positive feedback mechanism to amplify the effects of HSF1 (Li *et al*., 2011, 2014a). AMPK has been implicated in carcinogenesis and the progression of several types of cancer (Dasgupta and Chhipa, 2016). Our results revealed another mechanism that could regulate the activity of HSF1. We found that in pancreatic cancer, the activation of AMPK was uniformly suppressed, and the low expression of p‐AMPK correlated with a poor prognosis in patients (Duan *et al*., 2017). The knockdown of AMPK in the pancreatic cancer cell lines elevated the expression of HSF1 and promoted the invasion and migration of cancer cells in an HSF1‐dependent manner (Fig. 6). However, the metformin‐induced AMPK activation suppressed the phosphorylation and nuclear translocation of HSF1. Therefore, we postulated that the loss of AMPK activation could be another mechanism that promotes the activation and amplification of HSF1.

The desmoplastic reaction, which is one of the most prominent histological features of PDAC, is characterized by dense stroma surrounding malignant epithelial cells due to the excessive production and deposition of the ECM, which is attributed to activated PSCs (Tang *et al*., 2013). The dense fibrotic stroma has been shown to promote the progression of pancreatic cancer (von Ahrens *et al*., 2017). We previously reported that in an orthotopic model of pancreatic cancer, a coinjection of PSCs with cancer cells significantly promoted tumor growth, metastasis, and perineural invasion (Li *et al*., 2014c). Previous studies have suggested that activated PSCs promote the invasion and metastasis of cancer cells in a paracrine manner via the secretion of soluble factors, such as stroma‐derived factor 1, hepatocyte growth factor, and other inflammatory factors (Cui *et al*., 2016; Li *et al*., 2012). Recent publications showed that stroma‐derived exosomes containing lactate, amino acid, lipid, and other intermediate substrates promoted cancer progression by reprogramming cancer cells to upregulate glycolysis and inhibit mitochondrial oxidative phosphorylation (Zhao *et al*., 2016). The fibrotic stroma can also support the maintenance of cancer cells by secreting alanine in an autophagy‐dependent pathway (Sousa *et al*., 2016). Here, we found a correlation between the activation of HSF1 and the desmoplastic reaction in pancreatic cancer. The *in vivo* inhibition of HSF1 reduced the deposition of Masson‐stained extracellular collagen and the marker of activated PSCs, α‐SMA. Given the roles of AMPK activation in desmoplasia suppression and HSF1 inhibition (Duan *et al*., 2017), we revealed that AMPK inactivation could be involved in the desmoplastic reaction by activating HSF1.

Cancer‐related inflammation has been found to be associated with the initiation and progression of cancer (Colotta *et al*., 2009). The dense stroma of pancreatic cancer is filled with large numbers of immune cells, including macrophages, neutrophils, MDSCs, regulatory T cells (Treg), and a few cytotoxic T cells (Ino *et al*., 2013). A previous study has revealed that patients with a higher neutrophil‐to‐lymphocyte ratio are expected to have a shorter overall survival (Cheng *et al*., 2015). The recruitment of neutrophils into the stroma promotes the progression of pancreatic cancer through the release of a variety and high amounts of biological active proteases. Neutrophil‐derived MMP9 is a potent angiogenic factor that promotes vascularization in a VEGF‐independent manner (Bausch *et al*., 2011). In addition, neutrophil helps establish the immunosuppressive microenvironment of pancreatic cancer (Chao *et al*., 2016). Macrophages are other important immune cells that mediate the progression of pancreatic cancer, and macrophages have been shown to promote distant metastasis by supporting tumor growth and activating myofibroblasts (Nielsen *et al*., 2016). The pharmacological inhibition of macrophages significantly inhibited metastasis formation and angiogenesis (Griesmann *et al*., 2016). Quiescent PSCs could be activated when cocultured with macrophages, indicating that macrophages play a profibrosis role in pancreatic cancer (Shi *et al*., 2014). Our results highlight the important role of AMPK and HSF1 in the inflammatory microenvironment of pancreatic cancer. The metformin‐mediated activation alleviated the recruitment of neutrophils and macrophages partially through the inhibition of HSF1. Considering the immunosuppressive role of neutrophils and macrophages, we analyzed and observed an increased number of CD3‐positive cytotoxic T cells in the stroma.

## 5 Conclusion

This study revealed that HSF1 promotes the invasion and metastasis of pancreatic cancer. In addition, HSF1 also helps in establishing the desmoplastic and immunosuppressive microenvironment. The abnormal activation of HSF1 could be mediated by the loss of AMPK activation, and our results revealed that the mechanism by which AMPK activation suppressed the progression of pancreatic cancer is HSF1 dependent.

## Author contributions

QM, KC, and ZM designed the experiments; KC, WQ, and JL carried out the majority of the experiments; ZJ and LC analyzed the data; KC, BY, and JC organized the figures; KC wrote the manuscript; and WD reviewed it. They were helped by LS, CZ, ML, and JM. All authors read and approved the final manuscript.

## Supporting information


**Fig. S1.** Metformin‐mediated AMPK activation inhibited the activity of HSF1 in Panc‐1 cells.
