# Supplementary figures and images for: Loss of AMPK activation promotes the invasion and metastasis of pancreatic cancer through an HSF1‐dependent pathway
Source: Mol Oncol. 2017 Aug 29;11(10):1475–92. doi: 10.1002/1878-0261.12116 (PMC5623818; doi:10.1002/1878-0261.12116)

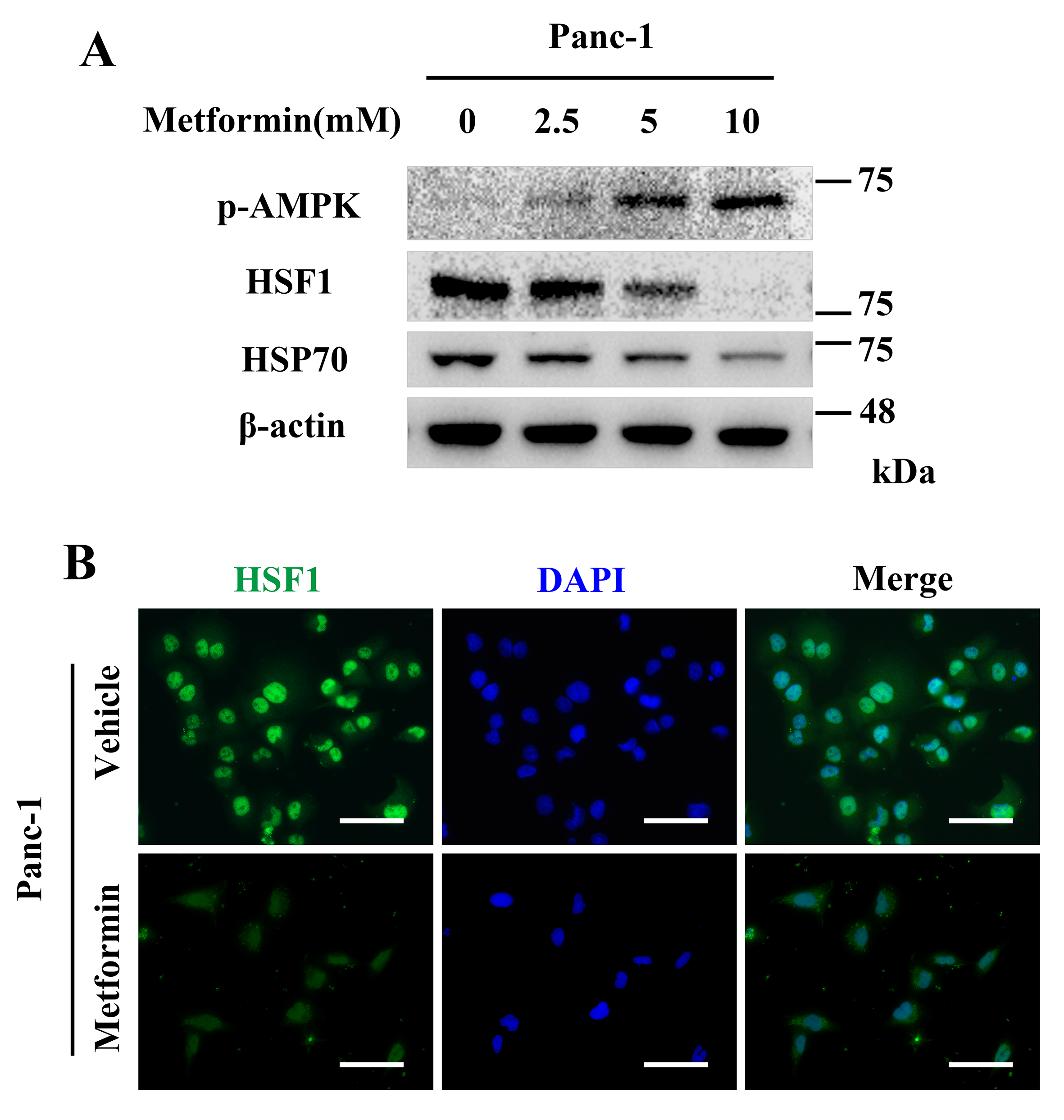

Supplement: Supplementary file 1 — Fig. S1. Metformin‐mediated AMPK activation inhibited the activity of HSF1 in Panc‐1 cells. [file MOL2-11-1475-s001.tif]
